# Determining the Innovativeness of Nurses Who Engage in Activities That Encourage Innovative Behaviors

**DOI:** 10.3390/nursrep14020066

**Published:** 2024-04-03

**Authors:** Marion Leary, George Demiris, J. Margo Brooks Carthon, Pamela Z. Cacchione, Subhash Aryal, Jose A. Bauermeister

**Affiliations:** 1 School of Nursing, University of Pennsylvania, Philadelphia, PA 19104, USA; gdemiris@nursing.upenn.edu (G.D.); jmbrooks@nursing.upenn.edu (J.M.B.C.); pamelaca@nursing.upenn.edu (P.Z.C.); bjose@nursing.upenn.edu (J.A.B.); 2 Leonard Davis Institute of Healthcare Economics, University of Pennsylvania, Philadelphia, PA 19104, USA; 3 Penn Presbyterian Medical Center, Philadelphia, PA 19104, USA; 4 School of Nursing, Johns Hopkins University, Baltimore, MD 21205, USA; biostat13579@gmail.com

**Keywords:** universal design, organizational innovation, healthcare reform, creativity

## Abstract

Background: We sought to understand the innovativeness of nurses engaging in innovative behaviors and quantify the associated characteristics that make nurses more able to innovate in practice. We first compared the innovativeness scores of our population; then we examined those who self-identified as an innovator versus those who did not to explore differences associated with innovativeness between these groups. Methods: A cross-sectional survey study of nurses in the US engaging in innovative behaviors was performed. We performed an exploratory factor analysis (EFA) to determine the correlates of innovative behavior. Results: Three-hundred and twenty-nine respondents completed the survey. Respondents who viewed themselves as innovators had greater exposure to HCD/DT workshops in the past year (55.8% vs. 36.6%, *p* = 0.02). The mean innovativeness score of our sample was 120.3 ± 11.2 out of a score of 140. The mean innovativeness score was higher for those who self-identified as an innovator compared with those who did not (121.3 ± 10.2 vs. 112.9 ± 14.8, *p* =< 0.001). The EFA created four factor groups: Factor 1 (risk aversion), Factor 2 (willingness to try new things), Factor 3 (creativity and originality) and Factor 4 (being challenged). Conclusion: Nurses who view themselves as innovators have higher innovativeness scores compared with those who do not. Multiple individual and organizational characteristics are associated with the innovativeness of nurses.

## 1. Introduction

Incorporating innovative behaviors in the profession of nursing is a relatively new notion, with most literature examining the use of innovation methodologies in practice dating back just 10–15 years [1,2]. In the last five years though, we have seen a groundswell of support encouraging individuals and organizations to consider incorporating innovation into education and practice [3,4,5,6,7]. This transformation has occurred because of the ever-changing healthcare environment that struggles with “wicked problems” (those that are notoriously difficult to solve) and emerging health concerns, as well as the need to understand and create new models of care and emerging technologies [3,8]. Because nurses are situated in the problem space with patients and communities, they can more deeply contextualize the problem areas and innovative solutions needed. But not all nurses see themselves as innovators or have the support to innovate. Therefore, determining what combination of individual and organizational characteristics enhances a nurse’s innovativeness will be key to nurses leading in this space.

Innovativeness is a personal characteristic defined as “a behavior which is dependent upon the perceived attributes of the innovation” [9]. It is also an awareness of the need to innovate [10]. Few studies within nursing have examined innovativeness [11]. There is still a paucity of data regarding what factors contribute specifically to the innovativeness of nurses and whether nurses are inherently innovative, as some have suggested, or if it is a behavior that can be developed, as called for by the Future of Nursing 2020–2030 report [5,11,12,13,14]. Educational institutions, programs for continuing education, and healthcare settings have a distinct opportunity to develop nurses as leaders in innovation, as fostering innovation in healthcare has the potential to improve outcomes for patients and health systems alike [15,16]. By understanding the characteristics of nurses engaging in innovative behaviors, including whether they self-identify as an innovator, health system leaders could use these data to assess and cultivate more balanced, innovative teams. However, without reliable data on the characteristics contributing to nurses’ innovativeness, it will not be possible to properly quantify and cultivate those innovative behaviors in practice [17].

The purpose of this study was to expand the limited findings in this area by examining the characteristics of nurses in the US who show interest in innovation and engage in activities that encourage innovative behavior. We sought to understand the innovativeness of these nurses and quantify the associated characteristics that make nurses more able to innovate in practice. We also sought to determine differences between those nurses who self-identified as innovators and those who did not. 

## 2. Materials and Methods

### 2.1. Study Design

This study was designed as a cross-sectional, observational survey study. We sought to measure participants’ innovativeness using the Scales for the Measurement of Innovativeness tool and examine their association with individual and organizational characteristics. Based on these results, we compared those who self-identified as innovators with those who did not to show similarities and differences related to their innovativeness scores. We hypothesized that there would be differences between the innovativeness scores and characteristics of those who identified as innovators the scores of those who did not; with those identifying as an innovator having higher innovativeness scores and possessing greater characteristics associated with innovativeness (e.g., attending more innovation events).

### 2.2. Setting

Data collection for this study began on 12 December 2022, and ended on 22 January 2023. The study population included nurses, and the sample specifically targeted nurses engaging in innovative behaviors. 

### 2.3. Partcipants

Eligible participants included nurses who engaged in innovative behaviors, were licensed, and worked in all settings in the US (e.g., clinical, educational, start-ups). Participants under 18 years of age, undergraduate nursing students, and nurses who do not read English were excluded from the study.

We sought to recruit a national sample of nurses who showed interest in innovation by engaging in activities supporting innovative behaviors in the last three years. Nurses were asked to complete the 10–15 min survey. No incentives to participate in the survey were offered. The snowballing technique was used, asking all participants to share the study description and survey link with their colleagues and on their social media platforms.

As noted, not all nurses have the opportunity to innovate. Therefore, to determine the characteristics that make a nurse more innovative, we sampled nurses who were actively engaging in innovative behaviors. Future work will look at comparing nurses engaging in innovative behaviors versus those not engaging in innovative behaviors to determine if they have different characteristics.

### 2.4. Variables

Participants’ innovative behavior, individual sociodemographic (e.g., age, race, gender, income, education, institutional setting, years of practice), and organizational data (e.g., hospital location, Magnet status, job satisfaction) were collected via self-report using the Qualtrics survey platform. Variables included in the demographic and organizational surveys were chosen based on the conceptual models informing this study: the Individual Innovation in the Workplace theory and the Diffusion of Innovation theory [18,19]. Some items in the demographic and organizational survey were modeled on the survey used in the 2016 RN4CAST to examine institutional changes regarding education, staffing, work environment, and well-being [20]. Permission was obtained from the author (MM) for use on this project.

### 2.5. Data Sources/Measurement

All questionnaires were distributed and completed by participants via the Qualtrics website (Provo, UT, USA). At the beginning of the survey, a screening tool was completed by the respondent. 

Once screening was completed, respondents who selected at least one innovative behavior were provided with study consent language. The participant consented by completing the survey and submitting it through the online portal (Qualtrics, Provo, UT, USA). 

#### 2.5.1. Scales for the Measurement of Innovativeness 

The Scales for the Measurement of Innovativeness score was used to assess nurses’ innovativeness. The original validated tool is a paper-based, self-report survey developed in 1977 to measure the willingness to change in an individual, not actual adoption behavior. This is an important distinction, as nurses may want to be innovative but may have constraints or barriers preventing them from innovating [9]. The Scales for the Measurement of Innovativeness has been used in previous studies to quantify the innovativeness of nurses [12,14]. Based on the Diffusion of Innovation theory, the scale was designed as a 7-point Likert scale [18]. The Scales for the Measurement of Innovativeness has a maximum score of 140 (20 questions ranked per the Likert scale 1–7); a higher score is associated with a higher degree of innovativeness and willingness to change [9]. We received permission from the publisher (Blackwell Publishing, Inc.) for this study to transfer the survey into an electronic format.

The Innovativeness score was calculated by scoring the 7-point Likert scale (Strongly Agree = 7, Agree = 6, Moderately Agree = 5, Undecided = 4, Moderately Disagree = 3, Disagree = 2, Strongly Disagree = 1) and totaling each of the individual respondents’ scores for the 20 questions included in the Scale. Some questions from the survey were reverse scored to account for the directionality of the wording of the question, as recommended by Hurt & Cook [9]. The total sum was calculated to obtain a total possible score of 140 for each respondent. 

#### 2.5.2. Innovative Behavior

The innovative behavior items included information on whether a respondent participated in any innovation events such as hackathons, accelerators, incubators, design sprints, design thinking workshops, human-centered design courses, innovation fellowships, and challenges, reported as yes/no; the frequency of participation (e.g., 1–2 times over the past three years) was also captured. Questions specific to a respondent’s ability or willingness to innovate were captured on a frequency scale of never to always or not at all to very.

#### 2.5.3. Individual Characteristics

Demographic data included age, race, gender, income, education, institutional setting, years of practice, clinical level, and specialty area. Questions were captured as continuous variables for age and years of practice; the remaining demographic questions were captured as categorical variables (e.g., for Race: Asian, Black, White, Other). Other individual characteristics captured included satisfaction with one’s current position as a nurse and institution, feeling supported, and number of years worked. The number of innovation events participated in and exposed to in the past year was also included.

#### 2.5.4. Organizational Characteristics

Organizational data included items such as hospital location (urban/rural) and Magnet status (yes/no). Other variables regarding perceived facilitators of and barriers to innovativeness were collected. Questions related to satisfaction with opportunities to be creative and innovative and to lead, as well as whether their institution offered any HCD/DT or Innovation education, lectures, resources, and workshops were also collected.

### 2.6. Statistical Methods

We estimated descriptive statistics (mean, standard deviation) for our variables of interest. We then examined whether participants’ individual and organizational characteristics differed based on whether a participant self-identified as an innovator. For these analyses, we compared groups using Student’s *t*-tests or one-way ANOVAs with Tukey post hoc comparisons for continuous variables and Pearson’s Chi-square tests for categorical variables. To adjust for Type I errors in multigroup comparisons we employed a Tukey post hoc comparison where appropriate [21,22]. We also used Pearson correlations to examine linear associations between continuous variables. We used complete case analysis as minimal missing data did not affect the models we used.

Using exploratory factor analysis (EFA), we confirmed the construct validity of the Scales for the Measurement of Innovativeness tool. Using Principal Axis Factoring with a varimax rotation, we assessed whether distinct factor scores would emerge with eigenvalue scores greater than one. The presence of distinct factor scores would suggest different components of what constitutes innovation according to the Scales for the Measurement of Innovativeness tool. After determining the extracted factors, we estimated the unique and shared explained variance across the 20 items. Consistent with best practices, factor loads greater than 0.4 indicated an item’s contribution to a defined factor during the analysis. Using bivariate comparisons, we created standardized (z-score) factor scores from the EFA and subsequently examined whether nurses’ individual and organizational characteristics differed across these factors.

### 2.7. Ethics Criteria

The study was approved by the University of Pennsylvania Institute Review Board (Protocol #852671).

## 3. Results

We received 662 survey responses. Of those, 305 (46.07%) began the screening survey but did not complete it. Three-hundred and forty-nine (52.72%) respondents passed the screening survey and agreed to participate, 4 (0.60%) completed the screening survey but subsequently chose not to participate and 2 (0.30%) completed the innovativeness survey but did not select that they agreed to participate and thus were removed from the analysis.

**Figure 1:**
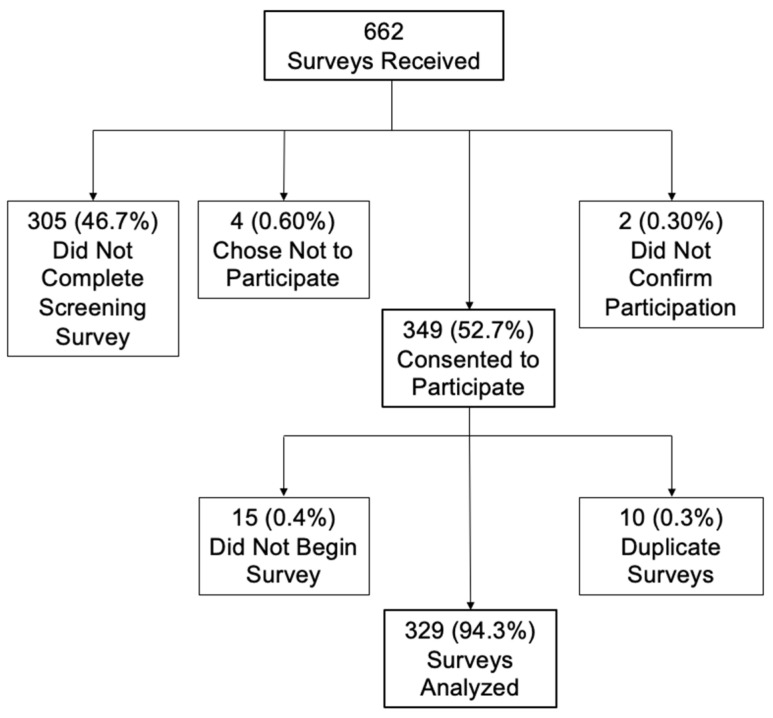
Enrollment schematic.

Of the 349 who passed the screening and agreed to participate, 15 did not start the survey and were removed. Ten respondents started the survey twice. Of the ten duplicate surveys, five were kept in the analysis. If the respondent had both an incomplete and complete survey, their incomplete survey was removed. If they had two completed surveys, their most recently completed were kept for analysis. A total of 329 surveys were analyzed (Figure 1).

### 3.1. Individual Characteristics

The mean age of respondents was 47.17 ± 12.19 years. Most respondents—264/305 (86.56%)—were female, and 234/305 (76.72%) were white (Table 1). Most respondents had either a master’s degree 122/306 (39.87%), or PhD or other Doctorate 87/306 (28.43%). The majority of respondents, 202/301 (67.11%), received their initial nursing education from a baccalaureate degree program (Table 1). On average, the respondents had worked in the nursing field for 21.49 ± 12.67 years. Less than half of the respondents, (129/300, 43.00%), worked in a hospital. The current positions of those who responded were quite diverse, with the most respondents in any category, 110/300 (36.67%), stating Other, which included positions such as “Founder”, “Entrepreneur”, “Consultant”, and “Educator”. 

Most respondents, 285/326 (87.42%), viewed themselves as innovators. The majority of the respondents had been exposed to HCD and DT activities 209/323 (64.70%), lectures 224/325 (68.82%), projects 199/324 (61.42%), workshops 174/324 (53.70%), and other related innovative activities 227/324 (70.06%). In the last year, most respondents participated in 1–3 innovation events 230/321 (71.65%) (Table 1).

### 3.2. Organizational Characteristics

Of those who worked in a hospital, 126/298 (42.28%) were at a hospital with Magnet status. Most respondents, 212/296 (71.62%), considered their institution innovative, and 219/296 (73.98%) considered their institution supportive of innovative thinking by its nurses. However, of those where it was applicable, only 56/135 (41.48%) got protected time away from the bedside to work on projects. Just over half of the nurses who responded, 176/323 (54.48%), were very willing to implement innovation methodologies in their day-to-day work, but just 121/323 (37.46%) and 28/323 (8.67%) felt as though they were often or always able to implement innovation methodologies in their day-to-day work (Table 2).

Few institutions offered resources 119/282 (42.20%), education 107/283 (37.81%), workshops 100/283 (35.34%), or lectures 102/283 (36.04%) specific to HCD and DT. Many institutions offered innovation resources 174/282 (61.70%), education 166/284 (58.45%), workshops 139/283 (49.12%), and lectures 156/285 (54.74%) (Table 2). 

### 3.3. Self-Identified as an Innovator

Differences in respondent characteristics based on whether the respondents viewed themselves as innovators (n = 285) or not (n = 41) showed no statistically significant difference in demographic characteristics (Table 1). However, we observed significant differences in participants’ self-identification as innovators. Respondents who self-identified as innovators had greater exposure to HCD/DT workshops in the past year (56.38% vs. 46.13%, *p* = 0.02). Additionally, those who self-identified as innovators were more willing to implement innovation methodologies in their day-to-day work (57.80% vs. 31.71%, *p* = 0.002), more satisfied with their opportunities to be creative (43.58% vs. 24.39%, *p* = 0.001) and their opportunities to be innovative (42.86% vs. 24.39% *p* = 0.002) (Table 2). 

### 3.4. Innovativeness Score 

The total sample’s mean innovativeness score was 120.3 ± 11.2 out of a score of 140. We found the mean difference in the total innovativeness score was higher for those who viewed themselves as innovators compared with those who did not (121.3 ± 10.2 vs. 112.9 ± 14.8, *p* =< 0.001) (Table 1). We also found the innovativeness scores to be higher for those who work in healthcare compared with those who do not (120.9±10.3 vs. 115.1±12.5, *p* = 0.003) and for respondents who had been exposed to HCD/DT workshops (121.5 ± 9.9 vs. 118.8 ± 12.5, *p* = 0.04) (Table 3). Additionally, a Student’s *t*-test showed differences in innovativeness scores regarding whether respondents felt their institutions supported innovative thinking by its nurses, (121.3 ± 9.6 vs. 117.7 ± 12.8, *p* = 0.01) and whether respondents’ institutions offered HCD/DT education (122.7 ± 9.2 vs. 118.9 ± 11.4, *p* = 0.003), lectures (122.8 ± 9.2 vs. 118.9 ± 11.3, *p* = 0.003), resources (122.6 ± 9.1 vs. 119.0 ± 10.8, *p* = 0.004), HCD/DT education (122.7 ± 9.2 vs. 118.9 ± 11.4, *p* = 0.003), lectures (122.8 ± 9.2 vs. 118.9 ± 11.3, *p* = 0.003), resources (122.6 ± 9.1 vs. 119.0 ± 10.8, *p* = 0.004), and workshops (122.2 ± 10.2 vs. 119.3 ± 10.9, *p* = 0.03) and innovation lectures (122.1 ± 9.6 vs. 118.1 ± 11.7, *p* = 0.002), and workshops(121.7 ± 10.0 vs. 118.9 ± 11.6, *p* = 0.03) (Table 4).

A one-way ANOVA was used to compare the innovativeness scores with how many innovation events a respondent participated in over the past year. We found that the more innovation events a respondent participated in, the higher that person’s innovativeness score would be (one event, 118.6 ± 11.2 vs. 2–3, 119.4 ± 10.8 vs. 4–5, 122.5 ± 12.8 vs. six or more, 125.0 ± 8.3, *p* = 0.004). Tukey’s test for multiple comparisons found a statistically significant difference in the innovativeness scores between the two groups; one innovation event compared with six or more innovation events (*p* = 0.006) and 2–3 events compared with six or more innovation events (*p* = 0.02) (Table 3).

### 3.5. Exploratory Factor Analysis

An EFA was performed. The total variance explained was 56.66% (Table 5). Four factors emerged in the rotated factor matrix, with Factor 1 having an eigenvalue of 6.38, which accounted for 31.88% of the variance. Factor 2 had an eigenvalue of 2.46, accounting for 12.29% of the variance. Factor 3 had an eigenvalue of 1.47, accounting for 7.37% of the variance. Factor 4 had an eigenvalue of 1.03, accounting for 5.13% of the variance (Table 6). 

### 3.6. Description of Factors

Factor 1 (risk aversion) included eight survey questions about risk aversion and reluctance to accept new ideas. Factor 2 (willingness to try new things and being an influencer and leader) included nine items related to survey questions that focused on willingness to try new things and being an influencer and leader in relation to new ideas. Factor 3 (creativity and originality) included three items focused on survey questions about creativity and originality in thinking and behavior. Factor 4 (being challenged) included two items related to survey questions focused on being challenged by unanswered questions and ambiguity. 

### 3.7. Comparison across the Factors

**Figure 2:**
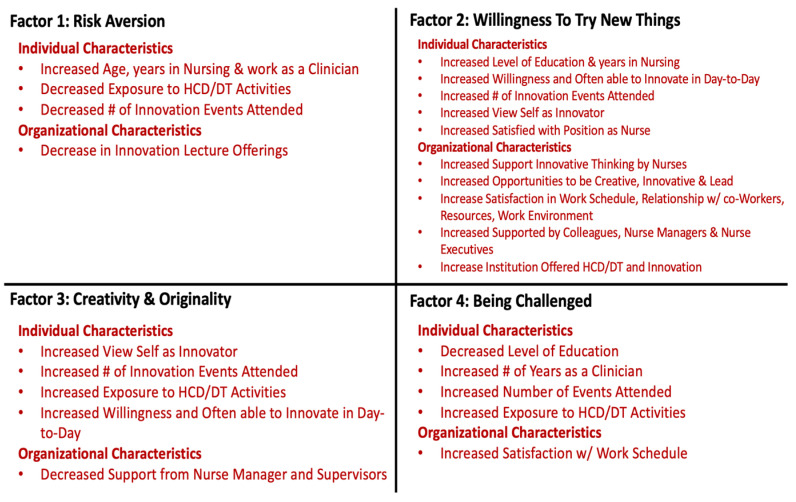
Correlates of innovative behavior.

We examined the associations between our four factors and the individual and organization characteristics (see Supplementary Material; Figure 2).

#### 3.7.1. Factor 1

For Factor 1 (risk aversion), A one-way ANOVA comparing how many innovation events respondents had participated in over the last year (*p* = 0.04), and their willingness to implement innovative methodologies in day-to-day work (*p* = 0.0001) were all positively associated with increased risk aversion. Though the one-way ANOVA showed a statistically significant difference in at least two groups for each variable, a Tukey test for multiple comparisons did not show differences in the pairwise analysis, respectively. A Student’s *t*-test found that whether respondents were exposed to HCD/DT activities in the last year or not was associated with risk aversion (*p* = 0.03). Additionally, a Student’s *t*-test found a difference in the level of risk aversion related to whether or not a respondent’s institution had offered innovation lectures in the past year (0.08 ± 0.78 vs. −0.15 ± 0.97, *p* = 0.03) (see Supplementary Material). 

#### 3.7.2. Factor 2

For Factor 2 (willingness to try new things and being an influencer and leader), a one-way ANOVA was performed and found a positive association with how many innovation events the respondent participated in over the last year (*p* =< 0.001), how often they could implement innovative methodologies in day-to-day work (*p* = 0.0006), and their willingness to implement innovative methodologies (*p* = 0.002). Though the one-way ANOVA showed a statistically significant difference in at least two groups for each variable, a Tukey test for multiple comparisons did not show differences in the pairwise analysis, respectively (see Supplementary Material).

A Student’s *t*-test found that whether respondents viewed themselves as innovators (0.05 ± 0.76 vs. −0.40 ± 1.3, *p* = 0.002) and whether their institutions supported innovative thinking by their nurses (0.08 ± 0.63 vs. −0.16 ± 1.0, *p* = 0.01) were associated with willingness to try new things and being an influencer and leader about new ideas.

Whether an institution offered innovation resources (12 ± 0.61 vs. −0.12 ± 0.94, *p* = 0.01), education (0.10 ± 0.64 vs. −0.008 ± 0.91, *p* = 0.05), workshops (0.12 ± 0.60 vs. −0.08 ± 0.89, *p* = 0.03), lectures (0.13 ± 0.61 vs. −0.11 ± 0.91, *p* = 0.01) and HCD/DT education (0.20 ± 0.55 vs. −0.08 ± 0.86, *p* = 0.003), lectures (0.18±0.56 vs. −0.07±0.85, *p* = 0.01), and resources (0.20 ± 0.56 vs. −0.07 ± 0.69, *p* = 0.001) were all positively associated with willingness to try new things and being an influencer and leader about new ideas.

#### 3.7.3. Factor 3

For Factor 3 (creativity and originality), a Student’s *t*-test showed that respondents were more willing to be creative and original in their thinking if they viewed themselves as innovators (0.11 ± 0.74 vs. −0.69 ± 1.0, *p* =< 0.001), and if in the last year, the respondent has been exposed to HCD/DT workshops (0.10 ± 0.68 vs. −0.09 ± 0.96, *p* = 0.04) and other HCD/DT events (0.21 ± 0.80 vs. −0.04 ± 0.80, *p* = 0.02). 

A one-way ANOVA found that how many innovation events the respondent had participated in over the last year (*p* = 0.01) was also positively associated with creativity and originality in thinking. Tukey’s test for multiple comparisons found a more significant difference in the Factor 3 score in one group regarding how many innovation events the respondent had participated in over the last year, six or more events compared with one event (*p* = 0.02) (Tables S1 and S2 in the Supplemental Material).

#### 3.7.4. Factor 4

For Factor 4 (being challenged), a one-way ANOVA found a significant difference in the respondents’ association with being challenged based on how many innovation events the respondents participated in over the last year (*p* =< 0.001). Though the Tukey test for multiple comparisons did not show differences in the pairwise analysis, the one-way ANOVA showed statistically significant differences in at least two groups for each variable (Tables S1 and S2 in the Supplemental Material).

## 4. Discussion

We were interested in understanding the innovativeness of nurses who engaged in innovative behaviors to quantify the characteristics and traits that make nurses more willing to be innovative in their practice. Understanding these will allow the tailoring of curricula for students, clinical education for nurses, and support work with health systems to create an environment more conducive to nurse-led innovation. Therefore, we sought to understand the individual and organizational characteristics of nurses who viewed themselves as innovators in comparison to those who did not, while also quantifying their innovativeness using the Scales for the Measurement of Innovativeness survey. 

We also performed an EFA to confirm the survey items’ contribution to one of the four identified factors: Risk Aversion (Factor 1), Willingness to Try New Things and Being an Influencer and Leader (Factor 2), Creativity and Originality (Factor 3) and Being Challenged (Factor 4). From the survey results and the EFA, we were able to identify the correlates of innovative behavior (Figure 2).

### 4.1. Individual and Organizational Characteristics

As noted, there was a paucity of data regarding the characteristics that contribute to the innovativeness of nurses. Our results showed that there are individual and organizational characteristics that contribute to nurses’ innovativeness. Nurses who viewed themselves as innovators had higher average innovativeness scores and were more satisfied with their opportunities to be creative and innovative at their institutions. 

Three main results emphasize these findings: (1) the number of innovation events participated in by respondents has a significant effect on their innovativeness scores, as those who attended six or more innovation events had higher innovativeness scores than those who attended 1–3 innovation events; (2) respondents who are more satisfied with aspects of their organizations had higher innovativeness scores than those who were not; and (3) respondents whose institutions offered various HCD/DT and innovation activities also had higher innovativeness scores compared with respondents whose institutions did not offer those activities. As noted, research focused on innovation has acknowledged specific individual and organizational characteristics that support innovative behavior; for individuals, a belief in one’s ability to be innovative and a focus on creativity; for organizations, supportive leadership as well as promoting awareness of and access to innovation [19,23,24].

Our findings showed that if nurses are exposed to innovation methodologies and activities, nurses are more willing to innovate. A recent study provided further evidence and found that nurses who attended innovation events, such as hackathons, were more confident when participating in innovative behaviors than those who had not [25].

Therefore, health system leaders invested in innovation should seek to create opportunities to encourage nurses to attend more HCD/DT and innovation events and offer these activities within their hospitals and health systems. Providing access to these may make nurses more willing to implement and create new ideas to solve the problems they see in their practices and be leaders in their institutions. Nevertheless, we found that only some institutions offered resources specific to HCD and DT. However, many offered resources specific to innovation. This is an important consideration, as we found in our previously published paper that nurses published papers regarding their use of HCD/DT methodologies in their practice less often than their physician counterparts [2]. Whether that means they are not using HCD/DT in practice or just not publishing their work needs further investigation. Regardless, future work should determine how hospitals and health systems define innovation, how they think about HCD and DT, and the rate at which they offer these activities to their nurses. 

The work environment plays a prominent role in the innovativeness of nurses. We found that respondents who worked at institutions that supported innovative thinking by their nurses had higher innovativeness scores than nurses who worked at institutions that they felt did not support innovative thinking by nurses. There was also a relationship between innovativeness scores, how often respondents were able to implement innovation in their day-to-day work, and how satisfied they were with their overall work environment. Organizations that support nurses in their ability to innovate foster nurses with higher innovativeness scores and who are more willing to innovate. However, in our population, very few respondents (8.6%) felt as though they could “always” implement innovation methodologies in their day-to-day work, even though the majority of respondents (54.5%) were “very” willing to innovate.

Additionally, many respondents “often” felt obstacles at their institutions impeded them from being innovative. This is a crucial point to consider, as one may be willing to innovate but lack the resources, structure, and institutional support to innovate. For example, most nurses in our survey who worked in a clinical role stated that they did not get protected time away from the bedside to work on other projects.

This is concerning. As noted, respondents whose organizations supported innovative thinking by their nurses had higher innovativeness scores. With the current nursing climate of burnout and understaffing, some institutions may not see the value in allowing their nurses time away from their clinical responsibilities to work on innovation projects or attend innovation and HCD/DT activities [26]. Institutions should consider the future ramifications of this type of thinking, as our results show that it could have a detrimental effect on the innovativeness of their nurses.

Moreover, how supported by a Nurse Manager a respondent felt was significantly different for those who viewed themselves as innovators than for those who did not, with those identifying as innovators feeling less supported. Leadership is essential in how supported nurses feel and their abilities to innovate. Hospital and health system leaders should consider addressing how they support their leadership through innovation to have a nursing staff that thinks differently and feels supported to transform care.

### 4.2. Scales for the Measurement of Innovativeness

To understand the innovativeness of nurses, we sought to determine the reliability and face validity of the Scales for the Measurement of Innovativeness tool in our population. We found that the Scales for the Measurement of Innovativeness survey was highly reliable in our population of nurses engaging in innovative behaviors. In addition, face validity suggests that respondents understood the questions being asked of them regarding their innovativeness and found the questions appropriate.

From our EFA, we discovered four domains to guide our contextualization of nurses’ innovativeness: Factor 1 included questions related to risk aversion and reluctance to accept new ideas. Factor 2 included questions connected with a willingness to try new things and being an influencer and leader in relation to new ideas. Factor 3 included questions regarding creativity and originality in thinking and behavior. Factor 4 focused on being challenged by unanswered questions and ambiguity.

Interestingly, counter to our previous results showing a positive relationship between participation at innovation events and innovativeness scores, there was a negative correlation between the number of innovation events respondents participated in and risk aversion and reluctance to accept new ideas for Factor 1, meaning that those who are risk averse and reluctant to accept new ideas participated in fewer innovation events. Therefore, encouraging nurses who may tend more toward risk aversion to attend events such as hackathons, where participants are encouraged to take risks and be creative in a safe and supportive environment, may help to decrease reluctance to innovate in their clinical practices. Future work should explore this hypothesis further.

The EFA also showed that, for nurses to be innovative, organizational factors play a significant role. Consequently, we need to consider how the environment of hospitals and health systems enables or dissuades nurses from leading in innovation. As was shown, nurses engaging in innovative behaviors are willing to innovate. However, if the environment is not conducive to encouraging innovation by nurses, innovative behaviors could be stifled.

### 4.3. Limitations

There are several limitations to this study. This study only surveyed nurses in the US; therefore, the heterogeneity of this group may not be generalizable to other nurses residing outside of the US. This was a cross-sectional study and, as such, may not represent the experiences of nurses over time. Building on this program of study, a longitudinal study should be considered in the future. Additionally, whether willingness to innovate translates to implementation needs to be determined.

As this study only examined nurses actively engaging in innovation behaviors that support innovation, we may have unintentionally excluded nurses who innovate in their clinical practice but have yet to participate in innovative behaviors such as hackathons, innovation workshops, or design sprints. Future studies should examine the innovativeness of all nurses, not just those engaging in innovative behaviors. This will allow us to understand the innovativeness of the nursing profession in general.

## 5. Conclusions

Compared to those who do not, nurses who view themselves as innovators have higher innovativeness scores. There are multiple individual and organizational characteristics that support the innovativeness of nurses, including how many innovation events one has participated in within the past year, exposure to HCD/DT workshops, whether one feels there is institutional support for innovative thinking by nurses, and whether the institution offered certain HCD/DT and innovation activities. Understanding how to incorporate these characteristics into the curricula of nursing schools and workplace culture will allow more nurses to be prepared to innovate and feel confident doing so. Academic institutions and healthcare organizations have a responsibility to support and foster the innovativeness of nurses for the good of the profession as well as the health and well-being of our patients and communities.

## Figures and Tables

**Table 1:** Individual characteristics by participant’s self-view as an innovator.

	Total Population	Views Self as an Innovator	Does Not View Self as an Innovator	t	df	*p*-Value
N	n = 329	n = 285	n = 41			
Age, years (m ± sd), n = 295	47.17 ± 12.18	47.69 ± 12.21	43.76 ± 11.62	1.88	1	0.06
Gender, n (%), n = 305				1.98	2	0.37
Female	264 (86.6)	226 (79.3)	38 (92.7)			
Male	33 (10.8)	30 (10.5)	3 (7.3)			
Other	8 (2.6)	8 (3.0)	0 (0)			
Race, n = 305				5.27	3	0.15
Asian	22 (7.2)	20 (7.0)	2 (4.9)			
Black	36 (11.8)	29 (10.1)	7 (17.1)			
Other	13 (4.3)	9 (3.2)	4 (9.8)			
White	234 (76.7)	206 (72.3)	28 (68.3)			
Hispanic or Latino, n = 306				0.61	1	0.66
Yes	15 (4.9)	14 (4.9)	1 (2.4)			
Highest Level of Education Completed in Nursing, n = 306				5.74	3	0.13
Hospital Diploma	0 (0)	0 (0)	0 (0)			
Associate Degree Program	0 (0)	0 (0)	0 (0)			
Baccalaureate Degree Program	52 (17.0)	46 (16.1)	6 (14.6)			
Master’s Degree	122 (39.9)	111 (39.0)	11 (26.8)			
Doctor of Nursing Practice	45 (14.7)	35 (12.3)	10 (24.4)			
PhD or other Doctorate	87 (28.4)	73 (25.6)	14 (34.2)			
Licensure, n = 306				3.75	3	0.29
LPN, RN	1 (0.3)	1 (0.4)	0 (0)			
RN	254 (83.0)	221 (77.5)	33 (80.4)			
Other	12 (3.9)	12 (4.2)	0 (0)			
RN, Other	39 (12.8)	31 (10.9)	8 (19.5)			
From What Type of Program Did You Receive Your Initial Nursing Education, n = 301				1.64	3	0.65
Associate Degree Program	49 (16.3)	42 (14.7)	7 (17.1)			
Baccalaureate Degree Program	202 (67.1)	172 (65.4)	30 (73.2)			
Diploma Program	23 (7.6)	21 (7.4)	2 (4.9)			
Graduate Program	27 (9.0)	25 (8.8)	2 (4.9)			
Current Position, n (%), n = 300				12.68	9	0.18
Staff Nurse	30 (10.0)	25 (8.8)	5 (12.2)			
Nurse Practitioner	19 (6.3)	16 (5.6)	2 (4.9)			
Clinical Nurse Specialist	12 (4.0)	8 (2.8)	4 (9.8)			
Nurse Anesthetist	1 (0.3)	0 (0)	1 (2.4)			
Nurse Midwife	2 (0.7)	1 (0.4)	1 (2.4)			
Nurse Manager	19 (6.3)	17 (6.0)	2 (4.9)			
Nurse Practitioner	19 (6.3)	16 (5.6)	3 (7.3)			
Senior Nursing Administrator	25 (8.3)	19 (6.7)	6 (14.6)			
Faculty member/Researcher	67 (22.3)	58 (20.3)	9 (22.0)			
Director of Innovation	15 (5.0)	15 (5.3)	0 (0)			
Other	110 (36.7)	99 (34.7)	11 (26.8)			
Are you satisfied with your current position as a nurse, n = 303				0.55	1	0.46
Yes	222 (73.3)	190 (66.7)	32 (78.1)			
No	81 (26.7)	72 (33.3)	9 (21.9)			
Are you satisfied with your current institution, n = 303				3.38	1	0.66
Yes	223 (73.6)	188 (66.0)	35 (85.4)			
No	80 (26.4)	74 (26.0)	6 (14.6)			
Do you feel supported by your						
Nurse Colleagues, n = 296	257 (86.8)	221 (77.5)	36 (87.8)	0.41	1	0.52
Nurse Manager, n = 288	217 (75.4)	182 (73.9)	35 (89.7)	5.03	1	0.03
Executive Leadership, n = 298	190 (63.8)	160 (56.8)	30 (73.2)	2.53	1	0.11
How many years have you worked in Nursing? (m ± stdev), n = 297	21.5 ± 12.7	21.9 ± 9	19.2 ± 1.8	1.25	1	0.21
How many years have you worked as a clinician? (m ± stdev), n = 294	14.6 ± 11.0	14.9 ± 0.7	12.8 ± 1.6	1.16	1	0.25
How many years have you worked at your current institution? (m ± stdev), n = 296	8.9 ± 9.0	8.8 ± 0.6	9.7 ± 1.4	−0.55	1	0.58
Do you work in a hospital?, n = 300				3.32	1	0.68
Yes	129 (43.0)	106 (37.2)	23 (56.1)			
No	171 (57.0)	153 (53.7)	18 (43.9)			
Employment Status, n = 301				2.94	2	0.23
Employed in healthcare	260 (86.4)	226 (79.3)	34 (82.9)			
Employed, but not in healthcare	33 (11.0)	26 (9.1)	7 (17.1)			
How many innovation events have you participated in the last 1 year? n = 321				4.18	3	0.24
1	111 (34.6)	93 (32.6)	18 (43.9)			
2–3	119 (37.1)	103 (36.1)	16 (39.0)			
4–5	46 (14.3)	41 (14.4)	5 (12.2)			
6 or more	45 (14.0)	43 (15.4)	2 (5.0)			
Have you been exposed to human-centered design/design thinking in the past 1 year	Yes					
Activities, n = 325	209 (64.3)	184 (64.6)	25 (61.0)	0.23	1	0.63
Lectures, n = 324	224 (69.1)	200 (70.2)	24 (58.5)	2.47	1	0.12
Other, n = 308	227 (73.7)	71 (24.9)	10 (24.4)	0.00	1	0.99
Projects, n = 324	199 (61.4)	177 (62.1)	22 (53.7)	1.19	1	0.28
Resources, n = 324	211 (65.1)	189 (66.3)	22 (53.7)	2.72	1	0.10
Workshops, n = 323	174 (53.9)	159 (56.4)	15 (46.1)	5.65	1	0.02
Innovativeness score, n = 329	120.3 ± 11.2	121.3 ± 10.2	112.9 ± 14.8	4.64	1	<0.001

PhD, doctor of philosophy; LPN, licensed practical nurse; RN, registered nurse.

**Table 2:** Organizational Characteristics by Participant’s Self-view as an Innovator.

	Total Population	Views Self as an Innovator	Does Not View Self as an Innovator	t	df	*p*-Value
N	n = 329	n = 285	n = 41			
Type of Institution, n (%), n = 298				6.44	3	0.09
Rural	20 (6.7)	19 (6.7)	1 (2.4)			
Suburban	61 (20.5)	53 (18.6)	8 (19.5)			
Urban	193 (64.8)	161 (56.5)	32 (78.1)			
Other	24 (8.1)	24 (0.4)	0 (0)			
Magnet Status, n = 298				3.06	2	0.22
Yes	126 (42.3)	106 (37.2)	20 (48.8)			
No	56 (18.8)	46 (16.1)	10 (24.4)			
Not Applicable	116 (38.9)	105 (36.8)	11 (26.8)			
Do you consider your institution to be innovative n = 296				0.01	1	0.92
Yes	212 (71.4)	183 (64.2)	29 (70.7)			
No	85 (28.6)	73 (25.6)	12 (29.3)			
Does your institution support innovative thinking by its nurses? n = 296				0.42	1	0.52
Yes	219 (74.0)	187 (65.6)	32 (78.0)			
No	77 (26.0)	32 (11.2)	9 (21.9)			
Do you get protected time away from the bedside to work on other projects? n = 294				.56	2	0.76
Yes	56 (19.0)	47 (16.5)	9 (22.0)			
No	79 (26.9)	67 (23.5)	12 (29.3)			
Not applicable	159 (54.1)	139 (48.8)	20 (48.8)			
How willing are you to implement innovation methodologies in your day-to-day work? n = 323				16.53	4	0.002
Not at all	1 (0.3)	0 (0)	1 (2.4)			
Rarely	3 (0.9)	2 (0.7)	1 (2.4)			
Somewhat	35 (10.8)	28 (9.8)	7 (17.1)			
Mostly	108 (33.4)	89 (31.2)	19 (46.3)			
Very	176 (54.5)	163 (57.8)	13 (31.7)			
How often are you able implement innovation methodologies in your day-to-day work? n = 324				8.58	4	0.07
Never	6 (1.9)	4 (1.4)	2 (4.9)			
Rarely	32 (9.9)	26 (9.1)	2 (4.9)			
Sometimes	137 (42.3)	115 (40.4)	22 (53.7)			
Often	121 (37.4)	112 (39.3)	9 (22.0)			
Always	28 (8.6)	26 (9.1)	2 (4.9)			
How often do you encounter obstacles that impede you from being innovative in your day-to-day work? n = 323				4.28	4	0.37
Never	1 (0.3)	1 (0.4)	0 (0)			
Rarely	25 (7.7)	23 (8.1)	2 (4.9)			
Sometimes	128 (38.6)	112 (39.3)	16 (39.0)			
Often	140 (43.3)	124 (43.5)	16 (39.0)			
Always	29 (9.0)	22 (7.7)	7 (17.1)			
How satisfied are you with the following aspects of your job: Opportunities for advancement, n = 296				2.01	3	0.57
Very Satisfied	104 (35.1)	90 (35.3)	14 (34.2)			
Moderately Satisfied	101 (34.1)	84 (32.9)	17 (41.2)			
A little Dissatisfied	52 (17.6)	45 (16.7)	7 (17.1)			
Very Dissatisfied	39 (13.2)	36 (14.1)	3 (7.3)			
How satisfied are you with the following aspects of your job: Opportunities to be creative,	n = 298			15.43	3	0.001
Very Satisfied	122 (10.9)	112 (43.6)	10 (24.4)			
Moderately Satisfied	93 (31.2)	73 (28.4)	20 (48.8)			
A little Dissatisfied	43 (31.2)	33 (12.8)	10 (24.4)			
Very Dissatisfied	40 (13.4)	39 (15.2)	1 (2.4)			
How satisfied are you with the following aspects of your job: Opportunities to be innovative,	n = 300			14.77	3	0.002
Very Satisfied	121 (40.3)	111 (42.9)	10 (24.4)			
Moderately Satisfied	89 (29.7)	69 (26.7)	20 (48.8)			
A little Dissatisfied	50 (16.7)	40 (15.4)	10 (24.4)			
Very Dissatisfied	40 (13.3)	39 (66.1)	1 (2.4)			
How satisfied are you with the following aspects of your job: Opportunities to lead,	n = 298			2.74	3	0.43
Very Satisfied	133 (44.6)	113 (44.0)	20 (48.8)			
Moderately Satisfied	98 (32.9)	84 (32.7)	14 (34.1)			
A little Dissatisfied	39 (13.0)	33 (12.8)	6 (14.6)			
Very Dissatisfied	28 (9.4)	27 (10.5)	1 (0.24)			
How satisfied are you with the following aspects of your job: Time away from clinical responsibilities,	n = 285			2.85	3	0.42
Very Satisfied	106 (37.2)	92 (37.2)	14 (36.8)			
Moderately Satisfied	76 (26.7)	66 (26.7)	10 (26.3)			
A little Dissatisfied	45 (15.8)	36 (14.6)	9 (23.7)			
Very Dissatisfied	58 (20.4)	53 (2.0)	5 (13.2)			
How satisfied are you with the following aspects of your job: Work schedule,	n = 299			2.47	3	0.48
Very Satisfied	168 (56.2)	145 (56.2)	23 (56.1)			
Moderately Satisfied	90 (30.1)	77 (29.9)	13 (31.7)			
A little Dissatisfied	29 (9.7)	27 (46.6)	2 (4.9)			
Very Dissatisfied	12 (4.0)	9 (32.1)	3 (7.3)			
How satisfied are you with the following aspects of your job: Choice of nursing as a career,	n = 305			1.60	3	0.66
Very Satisfied	208 (68.2)	179 (67.8)	29 (70.7)			
Moderately Satisfied	76 (24.9)	65 (24.6)	11 (26.8)			
A little Dissatisfied	16 (5.3)	15 (5.7)	1 (2.4)			
Very Dissatisfied	5 (1.6)	5 (1.9)	0 (0)			
How would you rate: Relationship with co-workers,	n = 298			1.38	3	0.71
Excellent	147 (49.3)	126 (49.0)	21 (51.2)			
Good	124 (41.6)	107 (41.6)	17 (41.5)			
Fair	19 (6.4)	16 (6.2)	3 (7.3)			
Poor	8 (2.7)	8 (3.1)	0 (0)			
How would you rate: Adequacy of resources,	n = 298			4.54	3	0.21
Excellent	95 (31.9)	77 (30.0)	18 (43.9)			
Good	135 (45.3)	117 (45.5)	18 (43.9)			
Fair	53 (17.8)	49 (19.1)	4 (9.8)			
Poor	15 (5.0)	14 (5.4)	1 (2.4)			
How would you rate: Support from supervisors,	n = 296			6.83	3	0.08
Excellent	128 (43.2)	104 (40.8)	24 (58.5)			
Good	96 (32.4)	83 (32.5)	13 (31.7)			
Fair	46 (15.5)	43 (16.9)	3 (7.3)			
Poor	26 (8.8)	25 (9.8)	1 (2.4)			
How would you rate: Overall work environment,	n = 298			1.24	3	0.74
Excellent	116 (38.9)	99 (38.5)	17 (41.5)			
Good	123 (41.3)	105 (40.9)	18 (43.9)			
Fair	54 (18.1)	49 (19.1)	5 (12.2)			
Poor	5 (1.7)	4 (1.6)	1 (2.4)			
Has your institution offered any of the following:	Yes					
HCD/DT education, n = 283	107 (37.8)	94 (33.0)	13 (31.7)	0.76	1	0.38
HCD/DT lectures, n = 283	102 (36.0)	90 (31.6)	12 (29.3)	0.96	1	0.33
HCD/DT resources, n = 282	119 (42.2)	106 (37.2)	13 (31.7)	1.80	1	0.18
HCD/DT workshops, n = 283	100 (35.3)	87 (30.5)	13 (31.7)	0.28	1	0.60
Innovation education, n = 284	166 (58.5)	145 (50.9)	21 (51.2)	1.03	1	0.31
Innovation lectures, n = 284	156 (54.9)	137 (48.1)	19 (46.3)	1.43	1	0.23
Innovation resources, n = 282	174 (61.7)	152 (53.3)	22 (53.7)	1.31	1	0.25
Innovation workshops, n = 283	139 (49.1)	120 (42.1)	19 (46.3)	0.15	1	0.70

HCD/DT, human-centered design/design thinking.

**Table 3:** Individual characteristics and innovativeness scores.

	Total Population	Innovativeness Score	t	df	*p*-Value
N	n = 329				
Age, yrs (m ± sd), n = 295	47.2 ± 12.2	r = 0.22			<0.001
Gender, n (%), n = 305			67.53	2, 302	0.43
Female	264 (86.6)	120.5 ± 10.2			
Male	33 (10.8)	118.4 ± 14.2			
Other	8 (2.6)	117.4 ± 12.8			
Race, n = 305			0.29	3, 301	0.41
Asian	22 (7.2)	118.7 ± 11.3			
Black	36 (11.8)	117.9 ± 10.2			
Other	13 (4.3)	119.2 ± 10.4			
White	234 (76.7)	120.8 ± 10.8			
Ethnicity, n = 306			−0.28	298	0.78
Hispanic or Latino	15 (4.9)	121.1 ± 12.5			
Highest Level of Education Completed in Nursing, n = 306			5.84	3, 302	0.60
Hospital Diploma	0 (0)	0 ± 0			
Associate Degree Program	0 (0)	0 ± 0			
Baccalaureate Degree Program	52 (17.0)	119.8 ± 10.4			
Master’s Degree	122 (39.9)	120.3 ± 9.7			
Doctor of Nursing Practice	45 (14.7)	122.0 ± 10.6			
PhD or other Doctorate	87 (28.4)	119.4 ± 12.3			
Licensure, n = 306			0.90	3, 302	0.53
LPN, RN	1 (0.3)	135 ± 0			
RN	254 (83.0)	120.1 ± 10.9			
Other	12 (3.9)	121.8 ± 9.2			
RN, Other	39 (12.8)	120.0 ± 10.0			
From What Type of Program Did You Receive Your Initial Nursing Education, n = 301			11.11	3, 297	0.91
Associate Degree Program	49 (16.3)	119.8 ± 13.3			
Baccalaureate Degree Program	202 (67.1)	120.1 ± 10.2			
Diploma Program	23 (7.6)	120.7 ± 11.7			
Graduate Program	27 (9.0)	121.5 ± 7.7			
Current Position, n = 300			17.29	9, 290	0.28
Staff Nurse	30 (10.0)	119.4 ± 11.9			
Nurse Practitioner	19 (6.3)	118.7 ± 11.0			
Clinical Nurse Specialist	12 (4.0)	117.4 ± 12.2			
Nurse Anesthetist	1 (0.3)	134 ± 0			
Nurse Midwife	2 (0.7)	108.5 ± 9.2			
Nurse Manager	19 (6.3)	118.2 ± 12.3			
Nurse Practitioner	19 (6.3)	118.7 ± 11.0			
Senior Nursing Administrator	25 (8.3)	119.5 ± 9.3			
Faculty member/Researcher	67 (22.3)	120.4 ± 11.7			
Director of Innovation	15 (5.0)	125.7 ± 7.7			
Other	110 (36.7)	121.0 ± 8.9			
Are you satisfied with your current position as a nurse, n = 305			−0.76	301	0.45
Yes	222 (73.3)	120.5 ± 10.3			
No	81 (26.7)	119.4 ± 12.0			
Are you satisfied with your current institution, n = 305			−0.59	301	0.55
Yes	223 (73.6)	120.4 ± 10.4			
No	80 (26.4)	119.6 ± 11.8			
Do you feel supported by your		Yes vs. No			
Nurse Colleagues, n = 296	257 (86.8)	120.8 ± 10.3 vs. 117.1 ± 13.2	−2.01	294	0.05
Nurse Manager, n = 288	217 (75.4)	120.3 ± 10.5 vs. 119.5 ± 11.9	−0.54	286	0.56
Executive Leadership, n = 298	190 (63.8)	120.7 ± 10.2 vs. 119.3 ± 11.5	−1.13	296	0.26
How many years have you worked in Nursing? (m ± stdev), n = 297	21.5 ± 12.7	r = 0.17	2.94	1	0.004
How many years have you worked as a clinician? n = 294	14.6 ± 11.0	r = 0.18	3.05	1	0.002
How many years have you worked at your current institution? n = 296	8.9 ± 9.0	r = 0.08	1.35	1	0.18
Do you work in a hospital? n = 300			1.10	298	0.27
Yes	129 (43)	120.9 ± 9.4			
No	171 (57)	119.6 ± 12.1			
Employment Status, n = 301			2.99	291	0.003
Employed in healthcare	260 (86.4)	120.9 ± 10.3			
Employed, but not in healthcare	33 (11.0)	115.1 ± 12.5			
How many innovation events have you participated in the last 1 year? n = 321			8.34	3, 317	0.004
1 ^a^	111 (34.6)	118.6 ± 11.2			
2–3 ^b^	119 (37.1)	119.4 ± 10.8			
4–5	46 (14.3)	122.5 ± 12.8			
6 or more	45 (14.0)	125.0 ± 8.3			
Have you been exposed to human-centered design/design thinking in the past 1 year:	Yes	Yes vs. No			
Activities, n = 325	209 (64.3)	121.4 ± 10.7 vs. 118.3 ± 11.9	−2.41	323	0.17
Lectures, n = 324	224 (69.1)	120.7 ± 12.4 vs. 119.3 ± 12.4	−0.97	322	0.33
Other, n = 308	227 (73.7)	122.5 ± 8.7 vs. 119.8 ± 11.3	−1.93	306	0.06
Projects, n = 324	199 (61.4)	120.7 ± 11.0 vs. 119.6 ± 11.6	−0.81	322	0.42
Resources, n = 324	211 (65.1)	121.0 ± 10.9 vs. 118.9 ± 11.7	−1.62	322	0.11
Workshops, n = 323	174 (53.9)	121.5 ± 9.9 vs. 118.8 ± 12.5	−2.11	321	0.04
Do you view yourself as an innovator? n = 326		Yes vs. No	−4.64	324	0.001
Yes	285 (87.4)	121.3 ± 10.2 vs. 112.9 ± 14.8			

Overall, there is a difference in the means but no difference in pairwise analysis; a Tukey test found differences in “How many innovation events have you participated in the last 1 year?” ^a^, 1 vs. 6 or more (*p* = 0.006) and ^b^, 2–3 vs. 6 or more (*p* = 0.02). Yrs, years; PhD, doctor of philosophy; LPN, licensed practical nurse; RN, registered nurse; m, mean; stdev, standard deviation.

**Table 4:** Organizational characteristics and innovativeness scores. (**A**) mean and standard deviation results; (**B**) correlation results.

**(A)**	**Total** **Population**	**Innovativeness Score (m/sd)**	**t**	**df**	***p*-Value**
	n = 329				
Type of Institution, n = 298			7.4	3, 294	0.10
Rural	20 (6.7)	121.8 ± 8.1			
Suburban	61 (20.5)	118.9 ± 12.3			
Urban	193 (64.8)	120.2 ± 10.5			
Other	24 (8.1)	125 ± 8.4			
Magnet Status, n = 298			0.35	180	0.72
Yes	126 (42.3)	119.2 ± 11.1			
No	56 (18.8)	119.8 ± 12.3			
Not Applicable	116 (38.9)	n/a			
Do you consider your institution to be innovative, n = 297			−1.29	295	0.20
Yes	212 (71.4)	120.9 ± 9.8			
No	85 (28.6)	119.1 ± 12.6			
Does your institution support innovative thinking by its nurses? n = 296			−2.58	294	0.01
Yes	219 (74.0)	121.3 ± 9.6			
No	77 (26.0)	117.7 ± 12.8			
Do you get protected time away from the bedside to work on other projects? n = 294			−0.99	133	0.33
Yes	56 (19.0)	120.8 ± 10.0			
No	79 (26.9)	118.8 ± 12.4			
Not applicable	159 (54.1)	n/a			
Has your institution offered any of the following:	Yes	Yes vs. No			
HCD/DT education, n = 283	107 (37.8)	122.7 ± 9.2 vs. 118.9 ± 11.4	−2.9672	281	0.003
HCD/DT lectures, n = 283	102 (36.0)	122.8 ± 9.2 vs. 118.9 ± 11.3	−2.9808	281	0.003
HCD/DT resources, n = 282	119 (42.2)	122.6 ± 9.1 vs. 119.0 ± 10.8	−2.9141	280	0.004
HCD/DT workshops, n = 283	100 (35.3)	122.2 ± 10.2 vs. 119.3 ± 10.9	−2.1655	281	0.03
Innovation education, n = 284	166 (58.5)	121.7 ± 10.0 vs. 118.3 ± 11.5	−2.6285	282	0.09
Innovation lectures, n = 284	156 (54.9)	122.1 ± 9.6 vs. 118.1 ± 11.7	−3.1240	282	0.002
Innovation resources, n = 282	174 (61.7)	121.1 ± 10.0 vs. 119.1 ± 11.8	−1.5120	280	0.13
Innovation workshops, n = 283	139 (49.1)	121.7 ± 10.0 vs. 118.9 ± 11.6	−2.1994	281	0.03
Other, n = 255	56 (22.0)	121.9 ± 9.8 vs. 119.7 ± 11.3	−1.3170	253	0.19
**(B)**	**Total** **Population**	**Innovativeness Score (Correlation Coefficient)**	***p*-Value**		
	n = 329				
How willing are you to implement innovation methodologies in your day-to-day work?	n = 323	r = 0.32	0.001		
Not at all	1 (0.3)				
Rarely	3 (0.9)				
Somewhat	35 (10.8)				
Mostly	108 (33.4)				
Very	176 (54.5)				
How often are you able implement innovation methodologies in your day-to-day work?	n = 324	r = 0.20	0.0003		
Never	6 (1.9)				
Rarely	32 (9.9)				
Sometimes	137 (42.3)				
Often	121 (37.4)				
Always	28 (8.6)				
How often do you encounter obstacles that impede you from being innovative in your day-to-day work?	n = 323	r = −0.09	0.13		
Never	1 (0.3)				
Rarely	25 (7.7)				
Sometimes	128 (38.6)				
Often	140 (43.3)				
Always	29 (9.0)				
How satisfied are you with the following aspects of your job: Opportunities for advancement	n = 296	r = 0.09	0.12		
Very Satisfied	104 (35.1)				
Moderately Satisfied	101 (34.1)				
A little Dissatisfied	52 (17.6)				
Very Dissatisfied	39 (13.2)				
How satisfied are you with the following aspects of your job: Opportunities to be creative	n = 298	r = 0.10	0.08		
Very Satisfied	122 (10.9)				
Moderately Satisfied	93 (31.2)				
A little Dissatisfied	43 (31.2)				
Very Dissatisfied	40 (13.4)				
How satisfied are you with the following aspects of your job: Opportunities to be innovative	n = 300	r = 0.10	0.09		
Very Satisfied	121 (40.3)				
Moderately Satisfied	89 (29.7)				
A little Dissatisfied	50 (16.7)				
Very Dissatisfied	40 (13.3)				
How satisfied are you with the following aspects of your job: Opportunities to lead,	n = 298	r = 0.08	0.16		
Very Satisfied	133 (44.6)				
Moderately Satisfied	98 (32.9)				
A little Dissatisfied	39 (13.0)				
Very Dissatisfied	28 (9.4)				
How satisfied are you with the following aspects of your job: Time away from clinical responsibilities	n = 285	r = 0.01	0.83		
Very Satisfied	106 (37.2)				
Moderately Satisfied	76 (26.7)				
A little Dissatisfied	45 (15.8)				
Very Dissatisfied	58 (20.4)				
How satisfied are you with the following aspects of your job: Work schedule	n = 299	r = 0.13	0.03		
Very Satisfied	168 (56.2)				
Moderately Satisfied	90 (30.1)				
A little Dissatisfied	29 (9.7)				
Very Dissatisfied	12 (4.0)				
How satisfied are you with the following aspects of your job: Choice of nursing as a career	n = 305	r = 0.02	0.02		
Very Satisfied	208 (68.2)				
Moderately Satisfied	76 (24.9)				
A little Dissatisfied	16 (5.3)				
Very Dissatisfied	5 (1.6)				
How would you rate: Relationship with co-workers, n = 298		r = 0.14	0.01		
Excellent	147 (49.3)				
Good	124 (41.6)				
Fair	19 (6.4)				
Poor	8 (2.7)				
How would you rate: Adequacy of resources, n = 298		r = 0.06	0.32		
Excellent	95 (31.9)				
Good	135 (45.3)				
Fair	53 (17.8)				
Poor	15 (5.0)				
How would you rate: Support from supervisors, n = 296		r = 0.07	0.25		
Excellent	128 (43.2)				
Good	96 (32.4)				
Fair	46 (15.5)				
Poor	26 (8.8)				
How would you rate: Overall work environment, n = 298		r = 0.12	0.04		
Excellent	116 (38.9)				
Good	123 (41.3)				
Fair	54 (18.1)				
Poor	5 (1.7)				

n, number; m/sd, mean/standard deviation; t, t-statistic; df, degrees of freedom; HCD/DT, human-centered design/design thinking; vs., versus.

**Table 5:** Total variance explained.

Total Variance Explained						
Factor	Initial Eigenvalues			Extraction Sums of Squared Loadings		
	Total	% of Variance	Cumulative %	Total	% of Variance	Cumulative %
1	6.38	31.88	31.88	5.87	29.37	29.37
2	2.46	12.29	44.17	1.93	9.64	39.01
3	1.47	7.37	51.54	1.09	5.46	44.47
4	1.03	5.13	56.66	0.54	2.68	47.15

**Table 6:** Exploratory factor analysis—rotated factor matrix.

n = 329		Factor			
	Mean (SD)	1 (Risk Aversion)	2 (Willingness to Try New Things)	3 (Creativity and Originality)	4 (Being Challenged)
I often find myself skeptical of new ideas. *	5.77 (1.06)	0.71			
I rarely trust new ideas until I can see whether the vast majority of people around me accept them. *	5.91 (1.05)	0.62			
I am reluctant about adopting new ways of doing things until I see them working for people around me. *	5.88 (1.16)	0.61			
I am aware that I am usually one of the last people in my group to accept something new. *	6.28 (0.95)	0.60			
I am suspicious of new inventions and new ways of thinking. *	5.76 (1.25)	0.60			
I must see other people using new innovations before I will consider them. *	5.98 (1.03)	0.59			
I tend to feel that the old way of living and doing things is the best way. *	6.01 (0.99)	0.49			
I am generally cautious about accepting new ideas.	4.59 (1.75)	0.43			
I enjoy trying out new ideas.	6.63 (0.73)		0.66		
I enjoy taking part in the leadership responsibilities of the groups I belong to.	6.24 (0.92)		0.62	.	
I seek out new ways to do things.	6.53 (0.78)		0.60		
I feel that I am an influential member of my peer group.	6.12 (0.99)		0.60		
My peers often ask me for advice or information.	6.36 (1.07)		0.56		
I am receptive to new ideas.	6.42 (0.77)		0.56		
I frequently improvise methods for solving a problem when an answer is not apparent.	6.10 (0.99)		0.46		
I am an inventive kind of person.	5.91 (1.14)			0.76	
I consider myself to be creative and original in my thinking and behavior.	6.19 (0.97)		0.43	0.65	
I find it stimulating to be original in my thinking and behavior.	6.40 (0.80)		0.46	0.46	
I am challenged by unanswered questions.	5.76 (1.40)				0.89
I am challenged by ambiguities and unsolved problems.	5.43 (1.70)				0.67

The items above were scored on a 7-point Likert scale ranging from 7–1; some items were reverse scored 1–7 as noted above with an asterisk (*).

## Data Availability

The data presented in this study are available on request from the corresponding author.

## Supplementary Materials

The following supporting information can be downloaded at: https://www.mdpi.com/article/10.3390/nursrep14020066/s1, Table S1: Exploratory Factor Analysis across Individual Characteristics; Table S2: Exploratory Factory Analysis across Organizational Characteristics.

## Public Involvement Statement

There was no public involvement in any aspect of this research.

## Guidelines and Standards Statement

This manuscript was drafted against the Strengthening the Reporting of Observational Studies in Epidemiology (STROBE) guidelines for reporting observational studies.
