# Supplementary material for: Determining the Innovativeness of Nurses Who Engage in Activities That Encourage Innovative Behaviors
Source: Nurs Rep. 2024 Apr 3;14(2):849–70. doi: 10.3390/nursrep14020066 (PMC11036237; doi:10.3390/nursrep14020066)
Supplement: Supplementary file 1 [file nursrep-14-00066-s001.zip › nursrep-2775883-supplementary.pdf]

Table S1. Exploratory Factor Analysis across Individual Characteristics.

	**Factor 1** (risk aversion)	**t**	**df**	**p-value**	**Factor 2** (willingness to try new things)	**t**	**df**	**p-value**
Age, n=295	r=0.19			0.001	r=0.11			0.07
Gender		1.27	295	0.21		0.63	295	0.53
Female, n=264	.02±0.87				0.02±0.66			
Male, n=33	-.19±1.1				-0.07±1.3			
Race		1.56	3, 301	0.81		6.42	3, 301	0.40
Asian, n=22	-0.16±0.80				0.15±0.52			
Black, n=36	-0.08±1.0				-0.11±0.68			
Other, n=13	-0.03±.095				-0.22±0.85			
White, n=234	0.01±0.89				0.03±0.78			
Hispanic		0.83	298	0.41		0.34	298	0.74
No, n=285	0.01±0.88				0.01±0.76			
Yes, n=15	-0.19±1.0				-0.06±0.77			
Highest Level of Education		3.09	3, 302	0.38		31.29	3, 302	<0.001*
Baccalaureate, n=52	0.07±0.91				-0.14±0.72			
Masters, n=122	-0.04±0.83				0.06±0.60			
DNP, n=45	0.11±0.93				0.07±0.63			
PhD, n=87	-0.10±0.98				0.01±1.0			
Licensure		0.06	304	0.95		-0.20	304	0.84
RN, n=254	-0.02±0.90				0.02±0.77			
Other, n=52	-0.01±0.93				-0.01±0.71			
From What Type of Program Did You Receive Your Initial Nursing Education		1.15	3, 297	0.92		42.12	3, 297	0.39
Associate, n=49	-0.01±0.95				-0.06±1.2			
Baccalaureate, n=202	-0.01±0.86				0.03±0.64			
Diploma, n=23	0.08±0.94				-0.02±0.71			
Graduate, n=27	-0.10±0.83				0.12±0.59			
Do you view self as an Innovator		-1.25	324	0.21		-3.20	324	0.002
Yes, n=285	0.02±0.87				0.05±0.76			
No, n=41	-0.17±1.05				-0.40±1.3			
Are you satisfied with your current position as a nurse		-0.21	301	0.83		-1.59	301	0.11
Yes, n=222	-0.01±0.92				0.06±0.64			
No, n=81	-0.03±0.87				-0.10±1.03			
Are you satisfied with your current institution		-0.05	301	0.96		-2.01	301	0.05
Yes, n=223	-0.02±0.89				0.07±0.64			
No, n=80	-0.02±0.93				-0.13±1.0			
Do you feel supported by your Nurse Colleagues		-0.97	294	0.34		-3.22	294	0.001
Yes, n=257	0.001±0.91				0.08±0.62			
No, n=39	-0.15±0.86				-0.34±1.3			

Do you feel supported by your Nurse Manager		-0.13	286	0.89		-2.33	286	0.02
Yes, n=217	-0.03±0.87				0.08±0.64			
No, n=71	-0.04±0.99				-0.17±1.1			
Do you feel supported by your Executive Leadership		-0.01	296	0.99		-2.41	296	0.02
Yes, n=190	-0.02±0.89				0.10±0.62			
No, n=108	-0.02±0.93				-0.12±0.95			
How Many Innovation Events Have you Participated in in the last year		8.59	3, 317	0.04*		54.41	3, 317	<0.001*
1, n=111	-0.08±0.98				-0.08±0.74			
2-3, n=119	-0.05±0.86				-0.01±0.87			
4-5, n=46	0.26±0.68				-0.05±1.3			
6 or more, n=45	0.15±0.79				.29±.42			
In the past 1 year, have you been exposed to human-centered design/design thinking Activities		-2.20	323	0.03		-0.57	323	0.57
Yes, n=224	-0.01±0.86				0.02±0.79			
No, n=100	0.01±0.98				-0.04±0.98			
In the past 1 year, have you been exposed to human-centered design/design thinking Lectures		0.24	322	0.81		-1.12	322	0.26
Yes, n=209	0.08±0.80				0.03±0.78			
No, n=116	-0.15±1.0				-0.08±1.0			
In the past 1 year, have you been exposed to human-centered design/design thinking Other		-1.44	306	0.15		-0.53	306	0.60
Yes, n=81	0.12±0.79				0.06±0.60			
No, n=227	-0.05±0.93				0.01±0.83			
In the past 1 year, have you been exposed to human-centered design/design thinking Projects		-0.54	322	0.59		-0.32	322	0.75
Yes, n=199	0.02±0.84				0.009±0.80			
No, n=125	-0.04±1.0				-0.02±0.96			
In the past 1 year, have you been exposed to human-centered design/design thinking Resources	0.04±0.85	-1.22	322	0.22	0.02±0.78	-0.56	322	0.57
Yes, n=211	-0.09±1.0				-0.04±0.99			
No, n=113								
In the past 1 year, have you been exposed to human-centered design/design thinking Workshops		-0.73	321	0.47		-0.88	321	0.38

Yes, n=174<br>No, n=149	0.03±0.81<br>-0.05±1.0				0.04±0.62<br>-0.05±1.1			
How many years have you worked in Nursing? n=297	r=0.13			0.02	r=0.14			0.02
How many years have you worked as a clinician? n=294	r=0.15			0.01	r=0.06			0.30
How many years have you been working at your current institution? n=296	r=0.06			0.32	r=0.06			0.05
Employment Status<br>Employed in healthcare, n=260<br>Employed but not in healthcare, n=33	 <br>.03±0.84<br>-.41±1.0	2.77	291	0.006	 <br>0.04±0.78<br>-0.09±0.64	0.89	291	0.38

*Overall, there is a difference in the means but no difference in pairwise analysis.

	**Factor 3** (creativity and originality)	**t**	**df**	**p-value**	**Factor 4** (being challenged)	**t**	**df**	**p-value**
Age, n=295	.r=0.10			0.07	r=0.07			0.27
Gender<br>Female, n=264<br>Male, n=33	 -0.03±0.78<br>0.24±1.0	-1.77	295	p=0.08	 0.01±0.88<br>-0.17±1.0	1.11	295	0.27
Race<br>Asian, n=22<br>Black, n=36<br>Other, n=13<br>White, n=234	 -0.12±0.95<br>-0.03±0.85<br>0.32±0.77<br>0.004±0.7	1.67	3, 301	0.64	 -0.23±0.90<br>-0.06±0.70<br>-0.14±1.1<br>0.04±0.91	4.85	3, 301	0.18
Hispanic<br>Yes, n=15<br>No, n=285	 0.29±0.70<br>-0.01±0.82	-1.42	298	0.16	 0.14±0.60<br>-0.002±0.91	-0.59	298	0.56
Highest Level of Education<br>Baccalaureate, n=52<br>Masters, n=122<br>DNP, n=45<br>PhD, n=87	 -0.024±0.83<br>0.02±0.85<br>0.09±0.65<br>-0.04±0.84	4.35	3, 302	0.23	 0.01±0.96<br>0.03±0.78<br>-0.11±0.99<br>-0.02±1.0	9.04	3, 203	0.03*
Licensure<br>RN, n=254<br>Other, n=52	 -0.01±0.80<br>0.09±0.86	0.75	304	0.45	 0.006±0.91<br>-0.09±0.93	-0.69	304	0.49
From What Type of Program Did You Receive Your Initial Nursing Education<br>Associate, n=49<br>Baccalaureate, n=202<br>Diploma, n=23<br>Graduate, n=27	 0.05±0.81<br>-0.06±0.81<br>0.22±0.75<br>0.19±0.84	0.35	3, 297	0.95	 0.05±0.81<br>-0.06±0.81<br>0.22±0.75<br>0.19±0.84	4.93	3, 297	0.18
Do you view self as an Innovator		-6.13	324	<0.001		-1.25	324	0.21

Yes, n=285<br>No, n=41	0.11±0.74<br>-0.69±1.0				0.02±0.90<br>-0.17±1.1			
Are you satisfied with your current position as a nurse<br>Yes, n=222<br>No, n=81	 <br>-0.02±0.79<br>0.08±0.86	0.93	301	0.36	 <br>-0.02±0.94<br>-0.02±0.86	0.003	301	0.99
Are you satisfied with your current institution<br>No, n=80<br>Yes, n=223	 <br>0.12±0.81<br>-0.03±0.81	1.44	301	0.15	 <br>-0.004±0.84<br>-0.02±0.95	0.14	301	0.89
Do you feel supported by your Nurse Colleagues<br>Yes, n=257<br>No, n=39	 <br>-0.03±0.78<br>0.22±0.99	1.78	294	0.08	 <br>0.01±0.88<br>-0.09±0.90	-0.64	294	0.52
Do you feel supported by your Nurse Manager<br>Yes, n=217<br>No, n=71	 <br>-.07±.81<br>.18±.83	2.18	286	0.03	 <br>-.02±.89<br>.04±.80	0.49	286	0.63
Do you feel supported by your Executive Leadership<br>Yes, n=190<br>No, n=108	 <br>-0.03±0.80<br>0.03±0.85	0.56	296	0.58	 <br>-0.03±0.93<br>0.03±0.82	0.51	296	0.61
How Many Innovation Events Have you Participated in in the last year, n=45<br>1, n=111<br>2-3, n=119<br>4-5, n=46<br>6 or more[a]	 <br><br>-0.18±0.91<br>0.08±0.76<br>0.04±0.88<br>0.25±0.59	12.04	3, 317	0.01	 <br><br>0.08±0.73<br>-0.16±1.1<br>0.04±0.86<br>0.18±0.80	18.10	3, 317	<0.001*
In the past 1 year, have you been exposed to human-centered design/design thinking Activities<br>Yes, n=224<br>No, n=100	 <br><br><br>0.08±0.72<br>-0.10±0.98	-1.84	323	0.07	 <br><br><br>0.04±0.91<br>-0.07±0.93	-1.00	323	0.32
In the past 1 year, have you been exposed to human-centered design/design thinking Lectures<br>Yes, n=209<br>No, n=116	 <br><br><br>-0.002±0.82<br>0.05±0.86	0.41	322	0.68	 <br><br><br>0.07±0.85<br>-0.17±1.1	-2.16	322	0.03
In the past 1 year, have you been exposed to human-centered design/design thinking Other<br>Yes, n=81<br>No, n=227	 <br><br><br>0.21±0.80<br>-0.04±0.80	-2.41	306	0.02	 <br><br><br>0.02±0.98<br>-0.001±0.90	-0.16	306	0.87

In the past 1 year, have you been exposed to human-centered design/design thinking Projects<br>Yes, n=199<br>No, n=125	 0.05±0.79<br>-0.06±0.88	-1.16	322	0.25	 -0.01±0.92<br>0.02±0.92	0.29	322	0.78
In the past 1 year, have you been exposed to human-centered design/design thinking Resources<br>Yes, n=211<br>No, n=113	 0.06±0.78<br>-0.09±0.90	-1.58	322	0.12	 -0.003±0.93<br>-0.001±0.90	0.02	322	0.99
In the past 1 year, have you been exposed to human-centered design/design thinking Workshops<br>Yes, n=174<br>No, n=149	 0.10±0.68<br>-0.09±0.96	-2.07	321	0.04	 0.08±0.86<br>-0.10±0.98	-1.80	321	0.07
How many years have you worked in Nursing?, n=297	r=0.01			0.93	r=0.09			0.15
How many years have you worked as a clinician?, n=294	r=0.06			0.28	r=0.12			0.05
How many years have you been working at your current institution? n=296	r=0.06			0.47	r=0.12			0.07
Employment Status<br>Employed in healthcare, n=260<br>Employed but not in healthcare, n=33	 0.02±0.80<br>-0.28±0.88	2.01	291	0.05	 0.001±0.91<br>0.09±0.73	-0.52	291	0.60

*Overall, there is a difference in the means but no difference in pairwise analysis; a, Tukey Test found differences in “How Many Innovation Events Have you Participated in in the last year” 6+ vs. 1 (p=0.02).

Table S2. Exploratory Factory Analysis across Organizational Characteristics.

	Factor 1 (risk aversion)	t	df	p-value	Factor 2 (willingness to try new things)	t	df	p-value
In what type of setting is your institution located		8.07	3, 294	0.05*		31.29	3, 294	<0.001*
Rural, n=20	0.06±0.93				0.07±0.60			
Suburban, n=61	-0.11±1.1				0.05±1.1			
Urban, n=193	-0.007±0.81				-0.01±0.67			
Other, n=24	0.19±0.80				0.21±0.52			
Do you work in a hospital		-0.02	298	0.98		0.94	298	0.35
Yes, n=129	-0.01±0.88				-0.03±0.94			
No, n=171	-0.01±0.87				0.06±0.58			
Magnet Status, if working in a hospital		1.53	180	0.13		-0.63	180	0.53
Yes, n=126	-0.12±0.91				0.01±0.68			
No, n=56	0.01±0.89				-0.07±1.1			
Do you consider your institution to be innovative		-1.16	295	0.25		-1.43	296	0.15
Yes, n=212	0.03±0.83				0.07±0.62			
No, n=85	-0.10±0.99				-0.07±1.0			
Does your institution support innovative thinking by its nurses?		-1.60	294	0.11		-2.47	294	0.01
Yes, n=219	0.04±0.84				0.08±0.63			
No, n=77	-0.15±0.96				-0.16±1.0			
Do you get protected time away from the bedside to work on other projects?		-0.09	133	0.93		-1.39	133	0.17
Yes, n=56	-0.06±0.88				0.11±0.61			
No, n=79	-0.07±0.97				-0.10±1.0			
How willing are you to implement innovative methodologies in your day-to-day work	r=0.29			0.0001	r=0.17			0.002*
Very, n=176								
Mostly, n=108								
Somewhat, n=35								
Rarely, n=3								
Not at all, n=1								
How often are you able to implement innovative methodologies in your day-to-day work	r=0.11			0.06	r=0.19			0.0006*
Always, n=28								
Often, n=121								
Sometimes, n=137								
Rarely, n=32								

Never, n=6								
How often do you encounter obstacles that impede you from being innovative in your day-to-day work?<br>Always, n=29<br>Often, n=140<br>Sometimes, n=128<br>Rarely, n=25<br>Never, n=1	r=-0.06			0.25	r=-0.05			0.38
How satisfied are you with your Independence at work<br>Very Satisfied, n=197<br>Moderately Satisfied, n=70<br>A little Dissatisfied, n=20<br>Very Dissatisfied, n=11	r=0.07			0.24	r=0.02			0.78
How satisfied are you with your Opportunities for advancement<br>Very Satisfied, n=104<br>Moderately Satisfied, n=101<br>A little Dissatisfied, n=52<br>Very Dissatisfied, n=39	r=0.06			0.33	r=0.99			0.09
How satisfied are you with your Opportunities to be creative<br>Very Satisfied, n=122<br>Moderately Satisfied, n=93<br>A little Dissatisfied, n=43<br>Very Dissatisfied, n=30	r=0.03			0.67	r=0.17			0.004
How satisfied are you with your Opportunities to be innovative<br>Very Satisfied, n=121<br>Moderately Satisfied, n=89<br>A little Dissatisfied, n=50<br>Very Dissatisfied, n=40	r=0.02			0.69	r=0.17			0.004
How satisfied are you with your Opportunities to lead<br>Very Satisfied, n=133<br>Moderately Satisfied, n=98<br>A little Dissatisfied, n=39<br>Very Dissatisfied, n=28	r=0.02			0.78	r=0.20			0.0005
How satisfied are you with your Time away from clinical responsibilities<br>Very Satisfied, n=106<br>Moderately Satisfied, n=76<br>A little Dissatisfied, n=45	r=-0.01			0.90	r=0.11			0.08

Very Dissatisfied, n=58								
How satisfied are you with your Work schedule<br>Very Satisfied, n=168<br>Moderately Satisfied, n=90<br>A little Dissatisfied, n=29<br>Very Dissatisfied, n=12	r=0.03			0.57	r=0.14			0.02
How would you rate the Relationship with co-workers<br>Excellent, n=147<br>Good, n=124<br>Fair, n=19<br>Poor, n=8	r=0.01			0.93	r=0.15			0.01
How would you rate the Adequacy of resources<br>Excellent, n=95<br>Good, n=135<br>Fair, n=53<br>Poor, n=15	r=0.01			0.93	r=0.15			0.01
How would you rate the Overall work environment<br>Excellent, n=116<br>Good, n=123<br>Fair, n=54<br>Poor, n=5	r=0.02			0.75	r=0.18			0.002
How would you rate Support from supervisors<br>Excellent, n=128<br>Good, n=96<br>Fair, n=46<br>Poor, n=26	r=-0.02			0.75	r=0.17			0.004
Has your institution offered any of the following innovation resources<br>Yes, n=174<br>No, n=108	 <br><br>-0.01±0.81<br>-0.04±0.99	-0.31	280	0.76	 <br><br>0.12±0.61<br>-0.12±0.94	-2.57	280	0.01
Has your institution offered any of the following innovation education<br>Yes, n=166<br>No, n=118	 <br><br>0.06±0.79<br>-0.12±0.97	-1.77	282	0.08	 <br><br>0.10±0.64<br>-0.08±0.91	-1.98	282	0.05
Has your institution offered any of the following innovation workshops<br>Yes, n=139<br>No, n=144	 <br><br>0.03±0.79<br>-0.06±0.95	-0.87	281	0.39	 <br><br>0.12±0.60<br>-0.08±0.89	-2.18	281	0.03
Has your institution offered any of the following Innovation lectures		-2.22	282	0.03		-2.64	282	0.01

Yes, n=156<br>No, n=128	0.08±0.78<br>-0.15±0.97				0.13±0.61<br>-0.11±0.91			
Has your institution offered any of the following HCD/DT education<br>Yes, n=107<br>No, n=176	0.11±0.74<br>-0.10±0.95	-1.99	291	0.05	0.20±0.55<br>-0.08±0.86	-3.02	281	0.003
Has your institution offered any of the following HCD/DT lectures<br>Yes, n=156<br>No, n=199	0.11±0.73<br>-0.09±0.94	-1.79	281	0.08	0.18±0.56<br>-0.07±0.85	-2.68	281	0.01
Has your institution offered any of the following HCD/DT resources<br>Yes, n=119<br>No, n=163	0.06±0.72<br>-0.09±0.96	-1.46	280	0.15	0.20±0.56<br>-0.07±0.69	-3.49	280	0.001
Has your institution offered any of the following HCD/DT workshops<br>Yes, n=100<br>No, n=183	0.07±0.75<br>-0.07±0.94	-1.29	281	0.20	0.14±0.62<br>-0.04±0.83	-1.86	281	0.06
Has your institution offered any of the following HCD/DT Other<br>Yes, n=56<br>No, n=199	0.13±0.77<br>-0.06±0.93	-1.37	253	0.17	0.19±0.55<br>-0.01±0.81	-1.77	253	0.08

	**Factor 3** (creativity and originality)	**t**	**df**	**p-value**	**Factor 4** (being challenged)	**t**	**df**	**p-value**
In what type of setting is your institution located<br>Rural, n=20<br>Suburban, n=61<br>Urban, n=193<br>Other, n=24	0.049±0.86<br>-0.25±0.97<br>0.03±0.76<br>0.38±0.56	10.58	3, 294	0.01	-0.08±1.0<br>0.01±0.85<br>-0.01±0.91<br>0.19±0.97	1.23	3, 294	0.75
Do you work in a hospital<br>Yes, n=129<br>No, n=171	-0.06±0.84<br>0.05±0.79	1.21	298	0.23	-0.10±0.99<br>0.09±0.83	1.84	298	0.07
Magnet Status, if working in a hospital<br>Yes, n=126<br>No, n=56	-0.06±0.77<br>-0.13±1.1	-0.46	180	0.65	-0.04±0.92<br>-0.08±0.88	-0.20	180	0.84
Do you consider your institution to be innovative<br>Yes, n=212	0.01±0.76	-0.09	295	0.93	-0.01±0.93	0.44	295	0.66

No, n=85	-0.002±0.94				0.04±0.85			
Does your institution support innovative thinking by its nurses?		0.09	294	0.93		-0.77	294	0.44
Yes, n=219	0.003±0.77				0.02±0.89			
No, n=77	0.01±0.95				-0.07±0.96			
Do you get protected time away from the bedside to work on other projects?		0.01	133	0.99		-1.06	133	0.29
Yes, n=56	-0.01±0.80				0.01±0.84			
No, n=79	-0.01±0.93				-0.17±1.0			
How willing are you to implement innovative methodologies in your day-to-day work<br>Very, n=176<br>Mostly, n=108<br>Somewhat, n=35<br>Rarely, n=3<br>Not at all, n=1	r=0.21			0.0002	r=-0.03			0.57
How often are you able to implement innovative methodologies in your day-to-day work<br>Always, n=28<br>Often, n=121<br>Sometimes, n=137<br>Rarely, n=32<br>Never, n=6	r=0.15			0.005	r=-0.05			=0.42
How often do you encounter obstacles that impede you from being innovative in your day-to-day work?<br>Always, n=29<br>Often, n=140<br>Sometimes, n=128<br>Rarely, n=25<br>Never, n=1	r=0.01			0.83	r=-0.05			0.36
How satisfied are you with your Independence at work<br>Very Satisfied, n=197<br>Moderately Satisfied, n=70<br>A little Dissatisfied, n=20<br>Very Dissatisfied, n=11	r=-0.11			0.06	r=0.07			0.26
How satisfied are you with your Opportunities for advancement<br>Very Satisfied, n=104<br>Moderately Satisfied, n=101	r=-0.05			0.35	r=0.0767			0.19

A little Dissatisfied, n=52<br>Very Dissatisfied, n=39								
How satisfied are you with your Opportunities to be creative<br>Very Satisfied, n=122<br>Moderately Satisfied, n=93<br>A little Dissatisfied, n=43<br>Very Dissatisfied, n=30	r=-0.02			0.72	r=0.032			0.58
How satisfied are you with your Opportunities to be innovative<br>Very Satisfied, n=121<br>Moderately Satisfied, n=89<br>A little Dissatisfied, n=50<br>Very Dissatisfied, n=40	r=0.02			0.80	r=-0.01			0.86
How satisfied are you with your Opportunities to lead<br>Very Satisfied, n=133<br>Moderately Satisfied, n=98<br>A little Dissatisfied, n=39<br>Very Dissatisfied, n=28	r=-0.10			0.08	r=0.002			0.98
How satisfied are you with your Time away from clinical responsibilities<br>Very Satisfied, n=106<br>Moderately Satisfied, n=76<br>A little Dissatisfied, n=45<br>Very Dissatisfied, n=58	r=-0.08			0.18	r=-0.01			0.88
How satisfied are you with your Work schedule<br>Very Satisfied, n=168<br>Moderately Satisfied, n=90<br>A little Dissatisfied, n=29<br>Very Dissatisfied, n=12	r=0.03			0.60	r=0.14			0.02
How would you rate the Relationship with co-workers<br>Excellent, n=147<br>Good, n=124<br>Fair, n=19<br>Poor, n=8	r=-0.06			0.29	r=0.02			0.76
How would you rate the Adequacy of resources<br>Excellent, n=95<br>Good, n=135<br>Fair, n=53<br>Poor, n=15	r=-0.06			0.29	r=0.02			0.76
How would you rate the Overall work environment				0.60				0.07

Excellent, n=116<br>Good, n=123<br>Fair, n=54<br>Poor, n=5	r=-0.03				r=0.11			
How would you rate Support from supervisors<br>Excellent, n=128<br>Good, n=96<br>Fair, n=46<br>Poor, n=26	r=-0.12			0.04	r=0.07			0.21
Has your institution offered any of the following innovation resources<br>Yes, n=174<br>No, n=108	-0.03±0.77<br>0.01±0.89	0.38	280	0.70	0.04±0.88<br>0.0002±0.87	-0.38	280	0.70
Has your institution offered any of the following innovation education<br>Yes, n=166<br>No, n=118	0.06±0.72<br>-0.11±0.93	-1.74	282	0.08	0.003±0.89<br>0.06±0.85	0.49	282	0.62
Has your institution offered any of the following innovation workshops<br>Yes, n=139<br>No, n=144	0.06±0.72<br>-0.09±0.90	-1.45	281	0.15	0.03±0.88<br>0.02±0.87	-0.11	281	0.91
Has your institution offered any of the following Innovation lectures<br>Yes, n=156<br>No, n=128	0.04±0.72<br>-0.08±0.92	-1.28	282	0.20	0.02±0.92<br>0.03±0.81	0.09	282	0.93
Has your institution offered any of the following HCD/DT education<br>Yes, n=107<br>No, n=176	0.05±0.76<br>-0.05±0.85	-0.91	281	0.37	-0.01±0.92<br>0.05±0.85	0.51	281	0.61
Has your institution offered any of the following HCD/DT lectures<br>Yes, n=156<br>No, n=199	0.08±0.72<br>-0.06±0.86	-1.45	281	0.15	0.02±0.88<br>0.03±0.87	0.02	281	0.98
Has your institution offered any of the following HCD/DT resources<br>Yes, n=119<br>No, n=163	0.04±0.75<br>-0.03±0.82	-0.74	280	0.46	0.03±0.92<br>0.04±0.82	0.13	280	0.90
Has your institution offered any of the following HCD/DT workshops<br>Yes, n=100	0.08±0.74	-1.38	281	0.17	-0.004±0.90	0.40	281	0.69

No, n=183	-0.06±0.85				0.04±0.86			
Has your institution offered any of the following HCD/DT Other		-0.07	253	0.95		1.35	253	0.18
Yes, n=56	-0.01±0.71				-0.14±1.0			
No, n=199	-0.03±0.84				0.04±0.85			

*Overall, there is a difference in the means but no difference in pairwise analysis; a Tukey Test found differences in “In what type of setting is your institution located” Other versus Suburban (p=0.007).
